# Improved social functioning and role functioning in rheumatic patients using a non-verbal communication tool: Results from a randomized, double-blind, controlled pilot-study

**DOI:** 10.3389/fmed.2023.1142350

**Published:** 2023-04-14

**Authors:** Julia Karnik, David Riedl, Michael Schirmer

**Affiliations:** ^1^Department of Internal Medicine, Clinic II, Medical University of Innsbruck, Innsbruck, Austria; ^2^Department of Psychiatry, Psychotherapy, Psychosomatics and Medical Psychology, University Hospital of Psychiatry II, Medical University of Innsbruck, Innsbruck, Austria; ^3^Ludwig Boltzmann Institute for Rehabilitation Research, Vienna, Austria

**Keywords:** communication, clinical trial, randomized, social interaction, rheumatic diseases, patient care, social fitness, EORTC QLQ-C30

## Abstract

**Introduction:**

Pain is a leading symptom in patients with rheumatic diseases, limiting not only physical functioning but also social well-being. This study studied the practicability of colored wristbands as non-verbal communication tools and the effects of these tools on social and role functioning in rheumatic patients.

**Methods:**

This prospective, double-blind, randomized controlled pilot study recruited 110 patients. Practicability of colored silicon wristbands as non-verbal communication tools was assessed by questionnaires. The control group received written information on the rheumatic diseases and their management in general. Social functioning and role functioning were assessed using two subscales from the EORTC QLQ-C30.

**Results:**

A significant overall improvement of social functioning (*p* = 0.005) and role functioning (*p* = 0.001) with medium to large effect size were reported by patients both in the intervention and the control group. *Post hoc* analyses revealed a significant change in the mean social functioning (*p* = 0.007) and role functioning scores with medium effect size, while no such effect was found in the control group for neither variable (*p* = 0.006 and *p* = 0.06–0.21, respectively). 42.9% of the patients will continue to use a non-verbal communication tool in the future. Practicability of the wristbands was limited by small size of the wristbands in 17.6% and uncomfortable wearing of the wristbands and skin irritation each in 4.4% of the patients.

**Discussion:**

This study shows first promising results for the use of a non-verbal communication tool in about 50.0% of the patients with rheumatic diseases, to improve their social functioning and role functioning.

## 1. Introduction

Pain is a leading symptom in patients with both inflammatory and non-inflammatory rheumatic diseases, limiting not only physical functioning but also social well-being (1). Chronic pain accompanied by limitations of activity and life participation was described as high impact chronic pain, which is clearly associated with a 2.4–4.1-fold higher risk for depression (2). Furthermore, patients with major depression have a 3-fold higher risk for suffering from non-neuropathic pain and a 6-fold higher risk to suffer from neuropathic pain (1). These limitations may then result not only in an emotional burden, but also in increased financial costs as an additional restriction to social wellbeing (3). Women are more likely to suffer from chronic pain, and this risk further increases with major depression, obesity, and a poor economic status (1, 2, 4). About two-thirds of patients with chronic pain are not satisfied with their treatment over years (3), although adequate pain treatment is considered a primary aim of treatment strategies (3). Nevertheless, in patients with rheumatoid arthritis (RA), chronic pain occurs more often than in patients without any immune-mediated rheumatic disease, especially if they are female (4). Overall, in RA chronic pain can be caused both by the disease itself and, independently from RA, by other non-inflammatory pain syndromes or fibromyalgia even if the underlying RA disease is sufficiently treated according to the treat-to-target principle (4).

From the patients’ perspective, anxiety and depression are not uncommon in patients with pain symptoms like in fibromyalgia. However, in 80% of these patients, these psychiatric symptoms occur even before the onset of fibromyalgia (5). Anxiety and depression were then related to increased perception of tenderness in tender points of fibromyalgia, but pain symptoms were also common in depressive patients without fibromyalgia, with higher pain thresholds and less avoidance of exposure to painful stimuli than fibromyalgia patients (5). Treatment options for the psychiatric comorbidities in fibromyalgia include cognitive behavior therapy, antidepressants, and physical exercise. Overall, chronic pain may then reduce social fitness and wellbeing.

In 1959 and again in 2002, the World Health Organization defined health positively, as complete physical, mental, and social well-being (6). In patients with rheumatic diseases, the Health assessment questionnaire (HAQ) is used to assess physical pain, function and health in general, but mental and social well-being are not routinely assessed (7). Thus, patients’ social fitness and well-being may be underrecognized by rheumatologists, as to the best of our knowledge there is no validated tool available to easily improve social fitness and well-being in patients with rheumatic diseases. Nevertheless, both social and role functioning have been routinely assessed in the large studies using specific dimensions of the SF-36 questionnaire (8). In oncological diseases, the EORTC QLQ-C30 is widely used (9).

This pilot study was performed to examine the practicability of colored silicon wristbands as a non-verbal communication tool for rheumatic patients, and to detect possible effects of this tool on the social functioning and role functioning of these patients.

## 2. Materials and methods

### 2.1. Study design, primary and secondary outcomes

The pilot study was designed as a prospective, double-blinded, randomized, controlled study. After informed and written consent, patients were recruited at a rheumatology outpatient clinic. Diagnoses were defined by rheumatologists based on the classification criteria.

Patients filled out a questionnaire at the beginning and the end of the study. The end of the study was defined as the next visit after study entry. At study entry, both patient groups received a closed box with the material as foreseen for the control and the intervention group. The physician and the patients were blinded to the assigned control or interventional group. Patients were routinely treated by their rheumatologist (SM). Outcome data were collected independently from the blinded investigator.

The primary outcome of this pilot study was to assess the practicability of colored silicon wristbands as a non-verbal communication tool. As a secondary outcome of the study, first experiences with the use and possible efficacy of the wristbands as a non-verbal tool of communication were assessed.

### 2.2. Study plan

Wristbands (from B&B Wristbands Ltd. Lincolnshire, GB, and Shenzhen Longhuaqu, Guangdongsheng, 518,000, China) were provided one in white color to each control patient and one each in white, green, yellow and red color to each patient in the intervention group. Costs were less than 4.95 € for each set of colored wristbands.

For the control group, the box included a general written information on their disease and treatment options together with a wristband (to be used as marker for study participation only outside the outpatient clinic). For the intervention group, the box included four wristbands in the different colors, together with written instructions and a calendar for documentation of their use. Instruction included their tasks (documented use of wristbands according to their current social fitness), how to clean the wristbands on a regular basis (at least once per day), and not to use the wristband within the rheumatology outpatient clinic to avoid unblinding for the rheumatologist. In the intervention group, the patients were encouraged to select family members, friends, working partners, etc. who are expected to fully respect the patient’s autonomy later when deciding on their actual social fitness in the future. Selected persons were then informed using written material provided to the patients. Patients and these informed persons should discuss consequences of the different colors when patients wear their wristbands.

For the intervention group, the white color was defined as perfect social fitness without pain (patient may even support others), green showed a very low level of pain (individual decision of possible activity to be made by the patient), yellow indicates pain but patient can fulfil daily work on his own (without need for help by others), and red indicates pain with need support for daily work. The patient was advised to define the meaning of the four colors for the different situations like work, daily life, sport, family, and others to be then explained to the family, friends and partners. These definitions should advise the family, friends, and partners how to react to each color in the appropriate way.

The control group received the same sized and weighted white package as the intervention group with different content. The control package contained one white wristband, a general information on the study together with four pages of information about RA, axSpA, PsA and vasculitis and collagenases.

### 2.3. Study size

There are no data available from previous studies concerning the sample size of an intervention in social well-being using a verbal or non-verbal communication tool. With an estimated type I error of *α* = 0.05; and a type II error of 1–*β* = 0.80 (*d* = 0.5), a study size of 102 patients was calculated. For the design with repeated measures of second endpoints the group size was calculated with a medium effect size of 98 patients (type I error = 0.05; Type II error = 0.80; *f* = 0.25). Therefore, the overall decision was made of a study size with 110 patients (55 each per intervention and control group), considering an estimated dropout rate of about 5–10%.

### 2.4. Inclusion and exclusion criteria for study participation

The case report form included age, sex, first symptom and evaluation of the diagnosis, clinical assessment of disease activity using the clinical disease activity index (CDAI), the clinical disease activity in psoriatic arthritis score (cDAPSA) and the Bath Ankylosing Spondylitis Disease activity index (BASDAI) as appropriate, and laboratory parameters like HLA-B27 status, levels of C-reactive protein (CRP), erythrocyte sedimentation rate (ESR) at study entry.

Inclusion criteria were age older than 17 years, estimated understanding of the tool, the questionnaires and the social contacts with family, friends, and other partners. Patients were included independent from gender and ethnicity. Exclusion criteria were visual loss or color blindness, severe psychiatric diseases, and a silicone allergy.

### 2.5. Randomization

Randomization was prepared using the sealed envelope generator (10). The patients were randomized to the interventional and the control group, with stratification according to their diagnoses into RA, axSpA, PsA, vasculitis or collagenoses and other non-inflammatory diseases. After filling up the prepared stratification lists, the patients were assigned to the empty slots as available.

### 2.6. Assessment of outcome parameters

Two slightly different versions were used for assessment of outcome parameters—one for the intervention and one for the control group. The questionnaire of the intervention group included 32 questions, concerning the practicability, handling, and helpfulness of the colored wristbands in the patient’s daily life as well as the reaction of the patient’s family, friends and partners about the non-verbal communication tool. The questionnaire for the control group included 14 different questions about the handling of the wristband and the usefulness of the information material. Both questionnaires included open questions, multiple choice questions for yes or no answers and a visual analog scale between 1 and 5, with higher scores indicating better outcomes. Furthermore, both questionnaires asked the patient if he or she talked about the study with friends and or family.

Social functioning and role functioning were assessed with two subscales from the EORTC QLQ-C30, which is a disease specific questionnaire to assess health-related quality of life and symptoms in patients with cancer (9). The QLQ-C30 is the most frequently used questionnaire to assess patient-reported outcomes in oncological trials (11). Two of the items assess social functioning and two other item’s role functioning. Items are scored on a four-point scale (0–3) and can be transformed into a 0–100-point scale, with higher scores indicating better levels of functioning.

### 2.7. Statistical considerations

After pseudonymization of the data set, statistical analyses were performed using the SPSS Statistics software (version 28.0.0; Armonk, NY, United States: IBM Corp).

Testing for normal distribution was performed using the Shapiro Wilk Test, as this test is more accurate for smaller sample sizes. Data were normally distributed with a *t*-value < 0.01. Descriptive statistics were then calculated for this normal distribution, with results depicted as median (with ranges in parentheses) or absolute numbers (with percentages in parentheses) as appropriate. For non-normally distributed data the Mann–Whitney-*U*-test was used for comparison of parameters between first and last visit. A *p* value < 0.01 is considered as highly significant, < 0.5 as significant, and < 0.2 as a trend towards significance. Nonparametric analyses were used because the data was not normally distributed.

To investigate mean change before and after the intervention in the primary outcome parameter (social functioning), a repeated measures analysis of variance (rANOVA) was calculated, with group assignment as grouping variable. Effect size values *η*^2^ = 0.01 were considered small, *η*^2^ = 0.06 as medium, and *η*^2^ = 0.14 as large (10). Bonferroni corrected *post-hoc* tests were calculated. Main analyses were conducted per-protocol. For sensitivity analysis, intention-to-treat analyses (last observation carried forward; LOCF) were conducted.

Concerning disease activity, ‘low disease activity’ and ‘remission’ where combined into one remission disease group. As the median age of the patients was 54 years, this number was chosen as cut-off to separate younger from older patients.

### 2.8. Ethical considerations

The study protocol was approved by the ethics committee of the Medical University of Innsbruck on July 21, 2021 (AN 1219/2021). The study was performed according to the Helsinki criteria, and patients were recruited only after informed and written consent. Data were pseudonymized for statistical analyses and anonymized prior to publication. Patients did not receive any payment or other kinds of rewards for participation in the study.

## 3. Results

### 3.1. Patients’ and diseases’ characteristics

A total of 87 out of the 110 recruited patients completed the study (38 from the intervention and 49 from the control group). The median days between study entry and the following visit with end of the study were 181 days with a range between 7 and 422 days, which depended on the need or the predefined appointment of a clinical visit. Out of the other 23 patients, 12 actively declined the last visit because of personal reasons (11 from the intervention and 1 from the control group), the other 11 patients were lost for follow-up. The study started during the Covid-19 pandemic and appointments were changed because of various lockdowns. Enrollment, allocation, follow-up and analysis are reported in the CONSORT flow chart in the Supplementary Material.

Overall, patients’ and diseases’ characteristics (Table 1), as well as the characteristics of the diagnostic groups (Table 2) did not differ between the control and the intervention groups. The group of patients with other inflammatory rheumatic diseases included patients with Sjögren’s syndrome, polymyalgia rheumatica/giant cell arteritis, systemic lupus erythematosus, and Behçet’s disease. Five patients had a non-inflammatory rheumatic disease like osteoarthritis.

**Table 1:** Patients’ and diseases’ characteristics at study entry.

	Control group (*n* = 55)	Intervention group (*n* = 55)	Total (*n* = 110)
Median age [years (range)]	54.0 (18–80)	54.0 (18–78)	53.5 (18–80)
Female (%)	78.2%	74.5%	76.4%
Time from 1st symptom to 1st visit [median months (range)]	155 (3–584)	124 (1–742)	141 (1–742)
Time from 1st symptom to diagnosis [median months (range)]	74.0 (0–584)	24.0 (0–480)	38.5 (0–584)
Time from diagnosis to 1st visit [median months (range)]	37 (0–636)	59 (0–634)	38 (0–636)
Clinical disease activity			
- Remission or low disease-activity (% of patients)	87.3%	87.3%	87.3%
- Moderate to high disease activity (% of patients)	12.7%	12.7%	12.7%
Laboratory data [median (range)]			
- ESR (mm/h)	9 (0–91)	10 (2–49)	10 (0–91)
- CRP (mg/dl)	0.34 (0.06–7.06)	0.18 (0.06–3.61)	0.20 (0.06–7.06)

CRP = C-reactive protein; ESR = erythrocyte sedimentation rate.

**Table 2:** Characteristics of disease groups at study entry (including laboratory data and assessments of disease activity with clinical scores as appropriate).

	Control group (*n* = 55)	Intervention group (*n* = 55)	Total (*n* = 110)
RA [*n* (%)]	13 (23.6%)	16 (29.1%)	29 (26.4%)
Rheumatoid factor [% +]	76.9%	81.3%	79.3%
ANA [% +]	46.2%	50.0%	48.3%
Median CDAI [VAS (range)]	6 (2–15)	3 (1–18)	4,5 (1–18)
axSpA [*n* (%)]	20 (36.4%)	16 (29.1%)	36 (32.7%)
HLA-B27 [% +]	55.0%	56.3%	55.6%
BASDAI [median (range)]	3,6 (0.2–8.1)	3.45 (2.2–6.6)	3.45 (0.2–8.1)
pSpA/PsA [*n* (%)]	12 (21.8%)	12 (21.8%)	24 (21.8%)
HLA-B27 [% +)]	25.0%	16.7%	20.8%
BASDAI [median (range)]	4.2 (2.8–5.2)	4.1 (0.7–6)	4.15 (0.7–6)
cDAPSA [median (range)]	6 (0–9)	8 (1–21)	6 (0–21)
Other inflammatory diagnosis [*n* (%)]	8 (14.5%)	8 (14.5%)	16 (14.5%)
Non-inflammatory diagnosis [*n* (%)]	2 (3.60%)	3 (5.50%)	5 (4.50%)

ANA = antinuclear antibodies; axSpA = axial spondyloarthritis; BASDAI = Bath ankylosing spondylitis activity index; CDAI = clinical disease activity index; pSpA = peripheral Spondyloarthritis; PsA = psoriatic arthritis; RA = rheumatoid arthritis; VAS = visual analog scale.

### 3.2. Primary outcome: Practicability of colored wristbands

The average patient used the wristbands 50.0% of the study period (ranging from 0 to 100%). 30.4% of the patients changed the color during the day. About 4.5% of the patients needed help to put on the silicon wristband.

For patients with RA, axSpA, pSpA/PsA or vasculitis/collagenosis, there was a trend that wristbands were worn more during active disease, compared to patients with low-disease activity or in disease remission (75 vs. 50%; *p* = 0.187). There was a trend that patients with active disease would have liked to use a non-verbal communication tool earlier in their disease course more than patients with low-disease activity or in remission (66.7 vs. 35.0%, respectively; *p* = 0.068).

Comprehension of information material was 80.7% in the intervention group. According to the patients’ perception, patients’ comprehension of information material appeared to be similar to comprehension of the information material prepared for friends, family and other partners. There was a trend, that comprehension of information material was better in younger patients (≤ 54 years) than in older patients (> 54 years; *p* = 0.122).

The main limitation was the small size of the wristbands in 17.6% of the patients. Adverse events of the silicone wristbands included skin irritation and uncomfortable wearing of the wristbands, and occurred in less than 5% of the control and the intervention group.

### 3.3. Secondary outcomes

The main secondary outcome results included social interactions with family friends and other partners, with an average of 5 persons who agreed to study participation per patients (ranging from 0 to 30 persons). The median age of these invited persons was 49.3 years (ranging from 10 to 85 years). 44.4% of the patients reported that their environment developed a highly positive attitude towards the patient’s disease.

#### 3.3.1. Scoring results for social functioning and role functioning

When combining both the intervention and the control groups, a significant overall improvement of social functioning (*p* = 0.005) and role functioning (*p* = 0.001) was detected with medium to large effect size (Table 3).

**Table 3:** Mean scores for social functioning and role functioning before and after intervention of the intervention group and in the control group.

		T1			T2			Time		Time*group	
		Mean	(SD)	p^T1^	Mean	(SD)	*p* ^T2^	*p*	*η* ^2^	*p*	*η* ^2^
Social functioning	Total (*n* = 67)	52.2	(30.7)		62.4	(31.6)		0.005	0.12	0.21	0.02
	Intervention (*n* = 29)	54.0	(34.7)	0.68	69.5	(30.6)	0.110	0.007	0.11		
	Control (*n* = 38)	50.9	(27.7)		57.0	(31.6)		0.21	0.02		
Role functioning	Total (*n* = 74)	38.1	(27.2)		49.1	(28.1)		0.001	0.14	0.40	0.01
	Intervention (*n* = 33)	42.4	(29.5)	0.22	56.6	(30.6)	0.040	0.006	0.10		
	Control (n = 41)	34.6	(25.1)		43.1	(24.7)		0.06	0.05		

T1 = study entry; T2 = end of study; SD = standard deviation; pT1 = level of significance between groups at T1; pT2 = level of significance between groups at T2; *η*^2^ = effect size: values of *η*^2^ = 0.01 were considered of small effect while *η*^2^ = 0.06 were defined as medium and *η*^2^ = 0.14 as large effect sizes.

**Figure 1:**
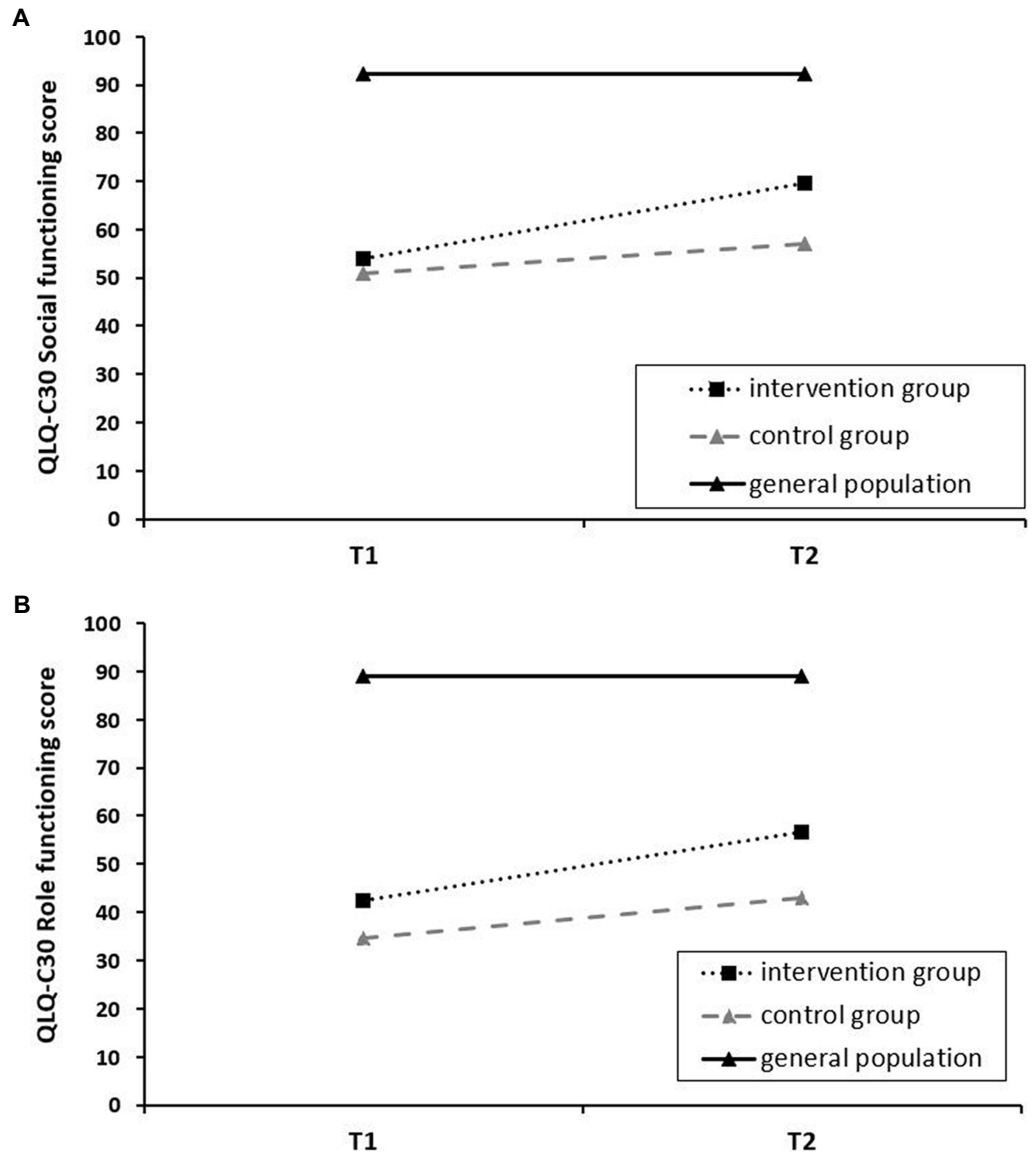
Mean scores for social functioning **(A)** and role functioning **(B)** at study entry (T1) and the end of the study (T2) for the intervention and control groups, according to the two subscales of the EORTC QLQ-C30. Study data were compared to data from the Austrian general population (9).

While no significant time*group effects were found for neither outcome, post-hoc analyses revealed a statistically significant change in the mean social functioning (*p* = 0.007) and role functioning score (*p* = 0.006) with medium effect size in the intervention group, while no such effect was found in the control group for neither variable (*p* = 0.06–0.21; as detailed in Table 3 and Figure 1).

Calculations were then repeated by imputing the last observation in case of missing data (intention to treat ITT; last observation carried forward = LOCF). Results remained stable for social functioning: while no significant difference was observed in the control group (*p* = 0.200), patients in the intervention group reported significantly improved social functioning (*p* = 0.013). For role functioning, patients in both groups reported significant improvements (intervention group: *p* = 0.010; control group: *p* = 0.049).

#### 3.3.2. Subjective assessments of tool’s efficacy

##### 3.3.2.1. Intervention group

Concerning the interaction between patients and their environment, 50.0% of the patients from the intervention group experienced the wristbands as helpful tools in their relationships, 41.7% would have liked to use a non-verbal communication tool earlier in their disease course, and 42.9% reported that they will continue to use a non-verbal communication tool in the future. 4.5% of the patients told their general practitioners about the study and their use of the wristbands.

Table 4 shows the subjective assessments of the wristbands on the patients’ life. There was a trend that patients under age of 54 years reported more impact of the non-verbal tool on their private life, sports’ activities and work life than the older patients. Nevertheless, both age groups rated the importance of the wristband with a median of 2.5 (ranging between 1 and 5).

**Table 4:** Age-dependent impacts of colored wristbands on private life, sports’ activities and work life (5 indicating the highest and 1 the lowest score of relevance).

	≤ 54 years	> 54 years	Total	*p*
Private life [median (range)]	3.0 (1–5)	2.0 (1–4)	3.0 (1–5)	0.095
Sports’ activities [median (range)]	2.5 (1–3)	1.0 (1–3)	2.0 (1–3)	0.070
Work life [median (range)]	3.0 (1–5)	1.0 (1–2)	1.5 (1–5)	0.020
Importance of wristband for patient [median (range)]	3.0 (1–5)	2.0 (1–4)	2.5 (1–5)	0.427
Improved conversations [median (range)]	1.0 (0–2)	1.5 (0–2)	2.0 (1–4)	0.857

Table 5 indicates differences of the subjective assessments of the wristbands on patients with low disease activity/remission and active disease. There was a trend that patients with an active disease during study intervention reported that the non-verbal tool was more important for them than reported by patients with remission or low disease activity (median/range: 4.0/2–5 versus 2.0/1–4). As shown in Table 6, work life differed significantly depending on age and disease activity, while the importance of the wristbands for the patients differed only depending on the disease activity.

**Table 5:** Disease activity-dependent subjective effects of colored wristbands on private life, sports’ activities and work life (5 indicating the highest and 1 the lowest score of relevance).

	Remission/low activity	Active disease	Total	*p*
Private life [median (range)]	3.0 (1–4)	3.5 (1–5)	3.0 (1–5)	0.188
Active (Sport) life [median (range)]	2.0 (1–3)	1.0 (1–1)	2.0 (1–3)	0.444
Work life [median (range)]	1.5 (1–3)	3.0 (1–5)	1.5 (1–5)	0.641
Importance of wristband for patient [median (range)]	2.0 (1–4)	4.0 (2–5)	2.5 (1–5)	0.146
Improved conversations [median (range)]	2.0 (0–2)	0.0 (0–0)	2.0 (1–4)	0.444

**Table 6:** Results of rANOVA-analysis of multiple independent factors.

	Age	Disease activity	Disease activity/age correlation
Private life	0.760	0.343	0.343
Sports’ activities	0.820	0.585	–
Work life	<0.001	0.041	0.024
Importance of wristband for patient	1.000	0.042	0.473
Improved conversations	0.234	0.627	0.807

A value of *p* < 0.05 indicates a significant result.

##### 3.3.2.2. Control group

The information material for the control group included short information on different diseases (e.g., on symptoms, etiology, therapeutic options). With 98% versus 80.7%, levels of comprehension of this information material was better in the control group compared to the information material on the wristbands in the intervention group (*p* = 0.008). This short disease-related information material was considered helpful with a median of 3 (ranging from 1 to 5).

## 4. Discussion

This study shows that a tool to improve the social aspect of WHO-defined health status can be considered as an interesting approach to be further studied, especially for patients less than 55 years of age and with an active inflammatory rheumatic disease. A non-verbal communication tool is practicable to improve the health condition in patients with specific rheumatic diagnoses, to be further evaluated as a voluntary add-on for improving the social aspect of health.

Overall, about half of the patients experienced the wristbands as helpful tools in their relationships, would have liked to use such a non-verbal communication tool even earlier, and reported that their social environment developed a highly positive attitude towards the patient’s disease. As a consequence, more than 40% of the patients want to continue to use colors for their non-verbal communication. On the other site, the drop-out rate of 21.9% was high, with many possible reasons: Every second of the dropout patients had no follow-up visit until the formal end of the study, whereas other reasons were the lack of any social contact, lack of pain or disease activity, change of rheumatologist and too complicated written information about the use of the wristbands. Especially the lack of any social contact appears to be important in the light of the significant difference between the patients’ and the scoring levels of the international control group [Figure 1, (9)], urging further attempts to improve social and role functioning in these patients.

This positive subjective assessment is highly encouraging to further evaluate such non-verbal communication approaches. Concerning the other secondary outcomes like effectiveness of such a communication tool there is still no final conclusion possible despite the blinded design of the study. It appears that younger patients and patients with more active disease preferred the wristbands more than elderly patients and those with low-disease activity or in remission. For a more systemic perspective of the patients’ needs, improvement of their social needs appears to be underestimated. Indeed, communication with the social environment using a non-verbal communication tool, led to improved social functioning and role functioning in the intervention group with medium effect size, but not in the control group. This indicates that wristbands have facilitated both social functioning and role functioning of patients in the intervention group. Other confounding factors possibly contributing to the improvement like disease activity have been considered, but without clear contribution.

Certainly, several issues have to be improved when used as a communication tool in the future. 17.6% of the patients claimed that the size of the wristband was either too tight or too loose. Individual sizes adapted to the patients’ handwrists are available and can help to solve this problem, as we provided only one standard size to each patient, independent from the patients’ size of the wrist. The few patients who do want to use the wristbands due to discomfort or skin irritations, may then use colored rings, t-shirts or hair bands as proposed by one creative patient, or stickers attached to their clothes as alternatives.

Second, the information to the patients has also to be improved, so that both the patients and their social environment better understand the potential and aims of such a non-verbal tool. Especially the consensus between patients and their social environment of what the colors mean to them is critical for the success of a non-verbal communication tool. We proposed specific aspects of life to be covered by the discussion of the colors with the patients’ social environment including general daily life, mobility, work life, sport activities, family and partnership. This discussion certainly needs to be done before the use of the wristbands, with personal introduction and explanation by health care workers to the patients. However, because of the blinded design, individual issues could be added but were not encouraged by the investigators. It can be assumed that personal explanation of the concept by unblinded health care workers or psychologists added to the written information will certainly further improve acceptance and comprehension of the information material especially in the patients older than 54 years. Therefore, the information material should certainly be adapted also to the age of the environment, which in this cohort strongly varied between 10 and 85 years. Beside such written information, personal explanations could help to understand the information.

The high appreciation of the written patients’ information on their diseases and therapeutic options by the control group shows the need for increased education of the patients. Besides, the control group did not receive any further active intervention. The worldwide web is full of information, but patients are often struggling with the abundance and reliability of this information (12). This study showed that a one-page paper sized information about the patient’s disease already helps the patients and their social environment a lot in understanding the diseases’ issues. Another limitation of the study was the lack of direct feedback from the social environment of the patients.

From the statistical perspective, unfortunately not all patients completed the questionnaires at the second assessment. The power of the secondary analyses, including evaluation of social functioning and role functioning, were therefore limited. To account for the lower number of patients with complete questionnaire data, an additional intention-to-treat (ITT) analysis (last observation carried forward) was conducted in addition to the per protocol evaluation. In this study, *post-hoc* tests were conducted although the omnibus test did not indicate a statistically significant interaction. While this is slightly unconventional, it has been reported that omnibus and *post-hoc* tests are not always in agreement, partially because they are testing different hypotheses (13). Since the omnibus test evaluates the overall pattern for any deviations among the means, it requires more statistical power than focused pairwise tests. Especially in studies with smaller samples, such as the current study, more subtle differences may be lost due to lack of power. Given the significant differences we found in our intervention group, but not in the control group, we would argue that in the case of this study the use of post-hoc tests in light of a non-significant omnibus-test were justified.

For the future, the ideal use of a non-verbal communication tool would be, that the physician or another health care provider explains the tool to the patient. The patient then receives written information and examples for the different traffic colors, to clarify the interpretation of the tools with their private and work environment. Further studies may focus on more patients with active disease, and may extend the use of this non-verbal communication tool to patients with other chronic diseases including especially oncological and pediatric diagnoses.

## Data availability statement

The data supporting the conclusions of this article will be made available by the authors on request.

## Ethics statement

The studies involving human participants were reviewed and approved by Ethics Committee of the Medical University of Innsbruck, Austria. The patients/participants provided their written informed consent to participate in this study.

## Author contributions

JK, MS, and DR contributed to the concept and design of the study. MS recruited and followed the patients. JK and DR performed the statistical analyses. JK, MS, and DR prepared the manuscript. All authors contributed to the article and approved the submitted version.

## Funding

The study was supported by the Verein zur Förderung der Hämatologie, Onkologie und Immunologie, 6020 Innsbruck, Austria.

## Conflict of interest

The authors declare that the research was conducted in the absence of any commercial or financial relationships that could be construed as a potential conflict of interest.

## Publisher’s note

All claims expressed in this article are solely those of the authors and do not necessarily represent those of their affiliated organizations, or those of the publisher, the editors and the reviewers. Any product that may be evaluated in this article, or claim that may be made by its manufacturer, is not guaranteed or endorsed by the publisher.
