# Supplementary figures and images for: Improved social functioning and role functioning in rheumatic patients using a non-verbal communication tool: Results from a randomized, double-blind, controlled pilot-study
Source: Front Med (Lausanne). 2023 Apr 14;10:1142350. doi: 10.3389/fmed.2023.1142350 (PMC10140413; doi:10.3389/fmed.2023.1142350)

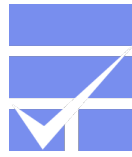

# CONSORT

TRANSPARENT REPORTING of TRIALS

## CONSORT 2010 Flow Diagram

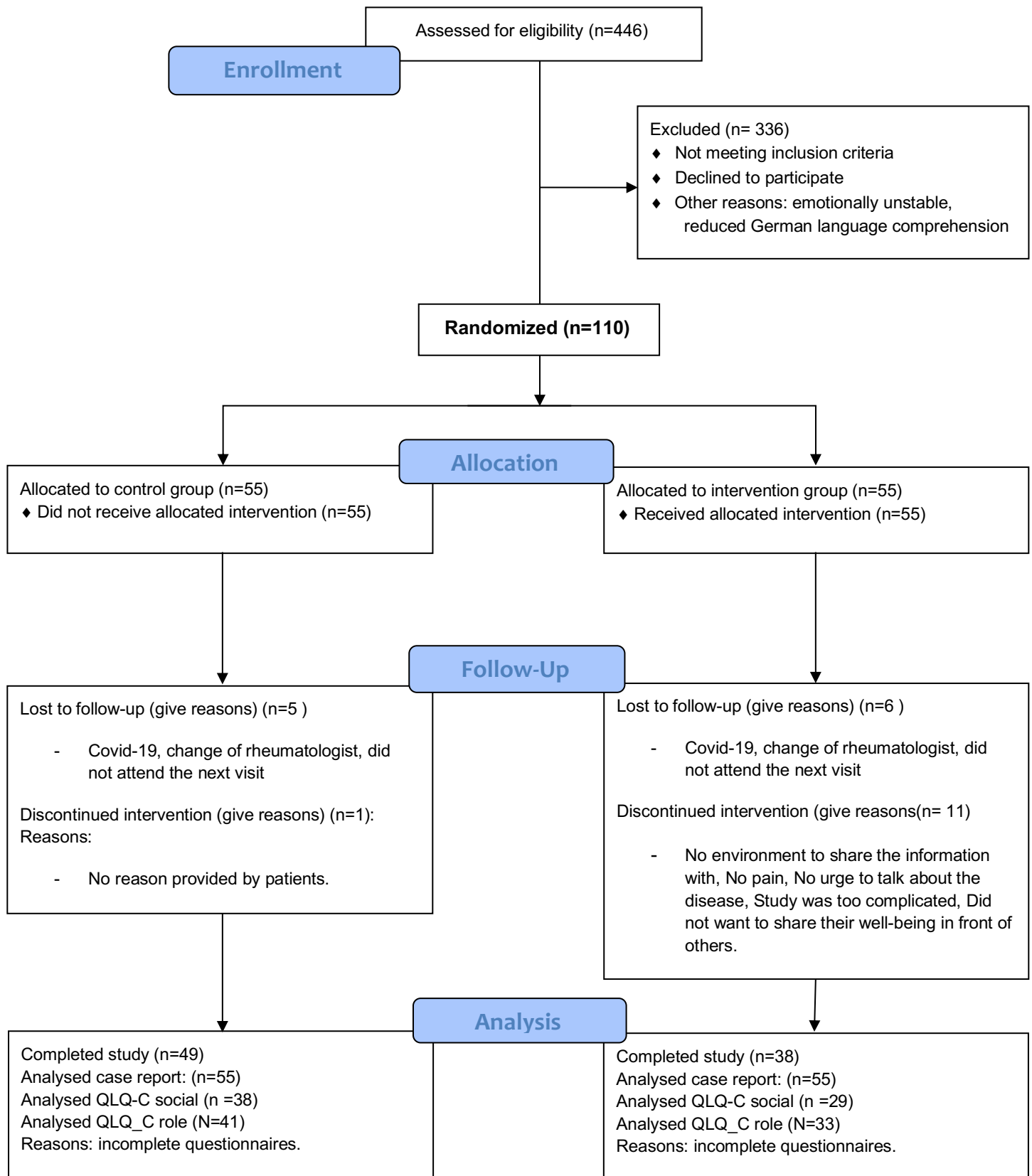

Supplement: Supplementary file 1 [file Presentation_1.PDF]
